# Synthesis, crystal structure, Hirshfeld surface and crystal void analysis of 4-fluoro­benzo[*c*][1,2,5]selena­diazol-1-ium chloride

**DOI:** 10.1107/S2056989025001379

**Published:** 2025-02-20

**Authors:** Atash V. Gurbanov, Tuncer Hökelek, Gunay Z. Mammadova, Khudayar I. Hasanov, Tahir A. Javadzade, Alebel N. Belay

**Affiliations:** a Excellence Center, Baku State University, Z. Xalilov Str. 23, Az 1148 Baku, Azerbaijan; b Hacettepe University, Department of Physics, 06800 Beytepe-Ankara, Türkiye; c Department of Chemistry, Baku State University, Z. Khalilov Str. 23, Az 1148 Baku, Azerbaijan; d Azerbaijan Medical University, Scientific Research Centre (SRC), A. Kasumzade Str. 14, AZ 1022 Baku, Azerbaijan; e Department of Chemistry and Chemical Engineering, Khazar University, Mahzati Str. 41, AZ 1096 Baku, Azerbaijan; f Department of Chemistry, Bahir Dar University, PO Box 79, Bahir Dar, Ethiopia; Vienna University of Technology, Austria

**Keywords:** crystal structure, non-covalent inter­actions, chalcogen bond, organic–inorganic salt

## Abstract

The asymmetric unit of the title salt, C_6_H_4_FN_2_Se^+^·Cl^−^, contains one planar 4-fluoro­benzo[*c*][1,2,5]selena­diazol-1-ium mol­ecular cation and a chloride anion. In the crystal, inter­molecular N—H⋯Cl hydrogen bonds link the 4-fluoro­benzo[*c*][1,2,5]selena­diazol-1-ium mol­ecules, which are arranged in parallel layers along (104), to the chloride anions. The cationic layers, in turn, are stacked along [001]. A Hirshfeld surface analysis of the crystal structure indicates that the most important contributions for the crystal packing are from H⋯Cl/Cl⋯H (22.6%), H⋯F/F⋯H (13.9%), H⋯N/N⋯H (11.9%), H⋯C/C⋯H (10.2%) and H⋯H (7.7%) inter­actions. The volume of the crystal voids and the percentage of free space were calculated to be 44.80 Å^3^ and 5.91%, showing that there is no large cavity in the crystal packing.

## Abstract 2

Charge-assisted chalcogen bonds with Se⋯Cl separations of 2.883 (2) and 3.030 (2) Å aggregate the title compound into a supra­molecular dimer.

## 1. Chemical context

Replacement of the H atom at the *R*—H⋯Nu synthon (Nu = nucleophile) with a group 16 element can lead to the formation of a chalcogen bond (ChB), which is a non-covalent inter­action between the electron-density-deficient side (so-called σ- or π-hole) of a covalently bonded chalcogen atom (Ch = O, S, Se or Te) and a nucleophilic (Nu) region in the same (intra­molecular) or another (inter­molecular) mol­ecular entity so that *R*—Ch⋯Nu [*R* = Ch, Pn (pnictogen), metal, *etc*.; Nu = lone pair possessing Ha (halogen), Ch, Pn or metal atom, anion, π-system, radical, etc.] can be formed (Aliyeva *et al.*, 2024 ▸). Similarly to hydrogen, halogen or pnictogen bonds, as well as to π-inter­actions (Abdelhamid *et al.*, 2011 ▸; Gurbanov *et al.*, 2018 ▸), the chalcogen bond is also of importance for the development of new catalysts based on metal complexes, or sensors, mol­ecular switches, *etc*. Following the concept of resonance-assisted hydrogen bonds (Maharramov *et al.*, 2010 ▸; Mahmudov *et al.*, 2011 ▸), a resonance-assisted chalcogen bond is usually treated as a chalcogen bond strengthened by conjugation in a π-system due to electron (charge) delocalization or favourable rearrangement of charge distribution in the mol­ecular system (Gurbanov *et al.*, 2020 ▸). Like charge-assisted hydrogen bonds (Mac Leod *et al.*, 2012 ▸; Martins *et al.*, 2017 ▸; Mizar *et al.*, 2012 ▸), the Ch⋯Nu bond can be strengthened by using an anion instead of traditional nucleophiles bearing a lone pair, which may lead to charge-assisted chalcogen-bonding (Guseinov *et al.*, 2022 ▸).
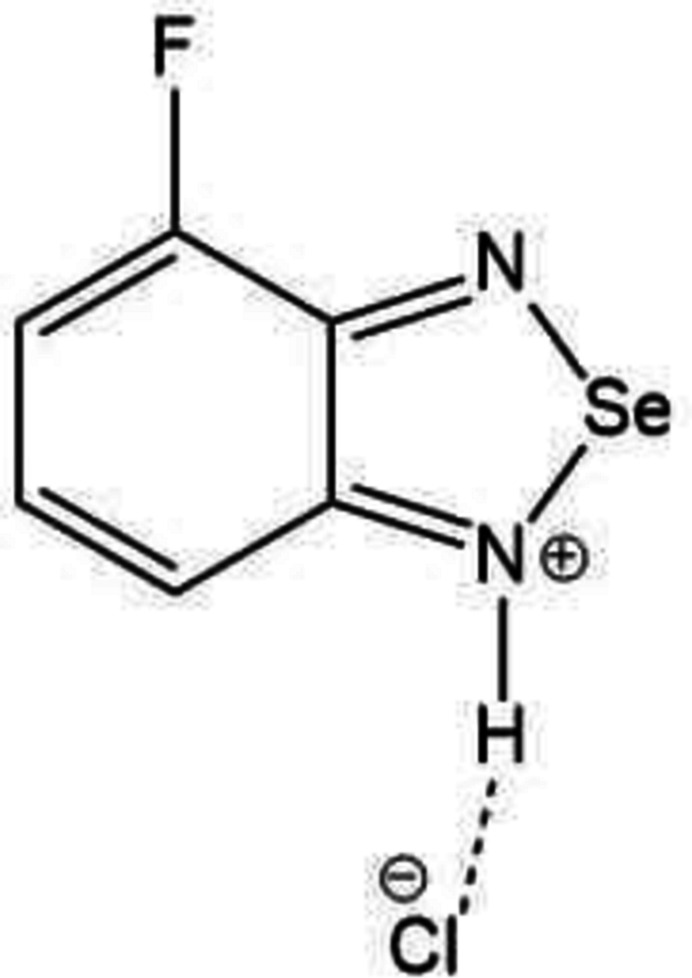


In the context given above, we have isolated the charge-assisted and chalcogen-bonded title salt, (C_6_H_4_FN_2_Se)^+^Cl^−^, and studied its mol­ecular and crystal structures together with a Hirshfeld surface and crystal voids analysis.

## 2. Structural commentary

**Figure 1:**
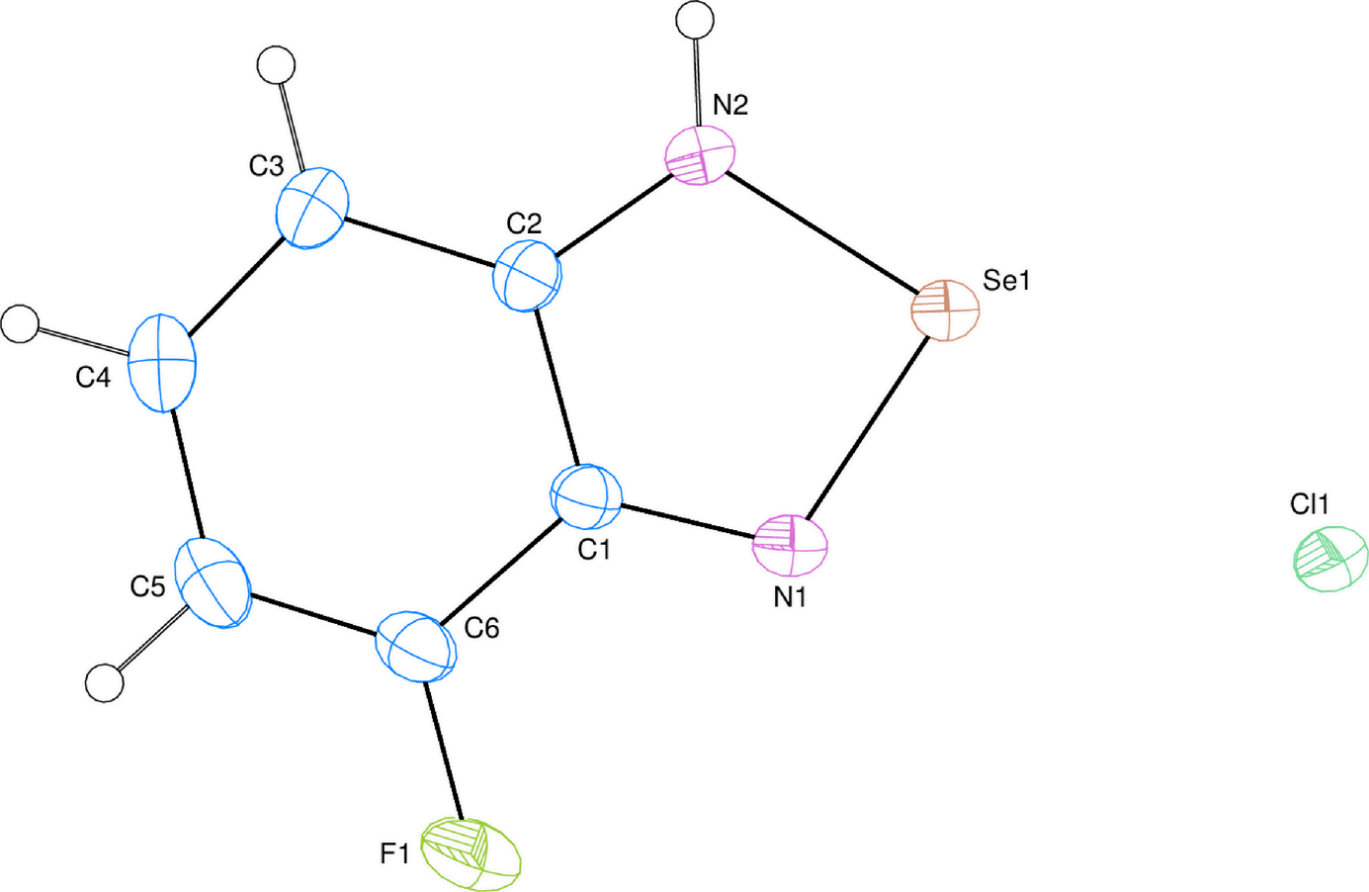
The asymmetric unit of the title compound with displacement ellipsoids drawn at the 50% probability level.

The asymmetric unit of the title compound contains one 4-fluoro­benzo[c][1,2,5]selena­diazol-1-ium cationic mol­ecule and a chloride anion (Fig. 1 ▸). The 4-fluoro­benzo[c][1,2,5]selena­diazol-1-ium mol­ecule is almost planar, where the planar *A* (C1–C6) and *B* (Se/N1/N2/C1/C2) rings are oriented at a dihedral angle of *A*/*B* = 0.64 (6)°. Atom F1 is 0.0063 (18) Å out of the least-squares plane of ring *A*. All bond lengths and angles in the mol­ecule are normal.

## 3. Supra­molecular features

**Figure 2:**
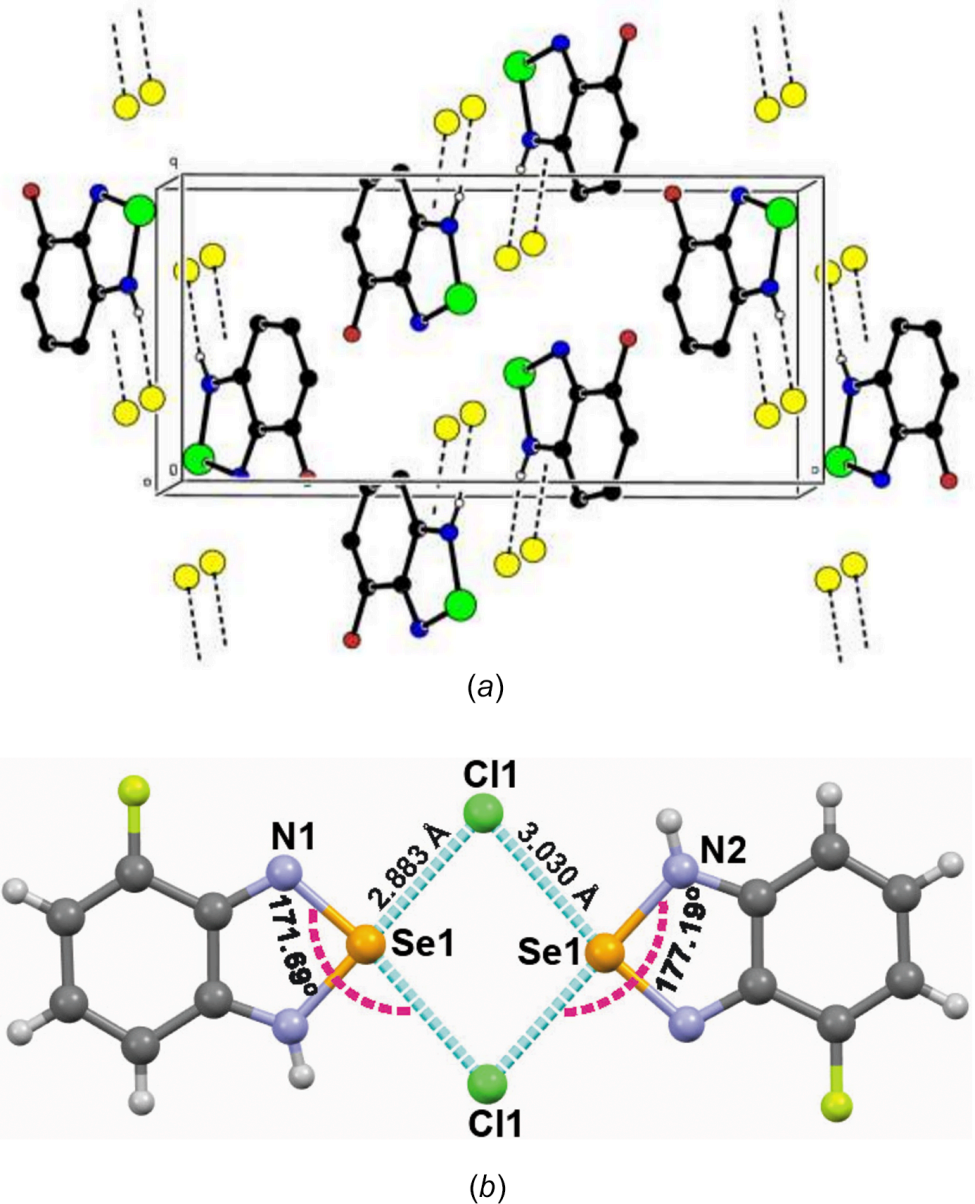
(*a*) Crystal packing diagram viewed down the *a* axis with inter­molecular N—H⋯Cl hydrogen bonds shown as dashed lines; (*b*) inter­molecular charge-assisted chalcogen bonds shown as dashed blue lines.

In the crystal, inter­molecular N—H⋯Cl hydrogen bonds link the mol­ecular cations, which are arranged into parallel layers along (104), and chloride ions (Table 1 ▸). The cationic layers, in turn, are stacked along [001 (Fig. 2 ▸*a*). The closest Se⋯Cl separations of 2.883 (2) and 3.030 (2) Å are shorter than the sum of the van der Waals radii (ΣrvdW (Se⋯Cl) = 3.65 Å) and therefore can be considered as charge-assisted chalcogen bonds, which aggregate the title compound into a supra­molecular dimer, with the σ-hole angles ∠N1—Se1⋯Cl1 and ∠N1—Se1⋯Cl1 of 171.69 (7)° and 177.19 (7)° (Fig. 2 ▸*b*). Neither π–π nor C—H⋯π(ring) inter­actions are observed.

## 4. Hirshfeld surface analysis

**Figure 3:**
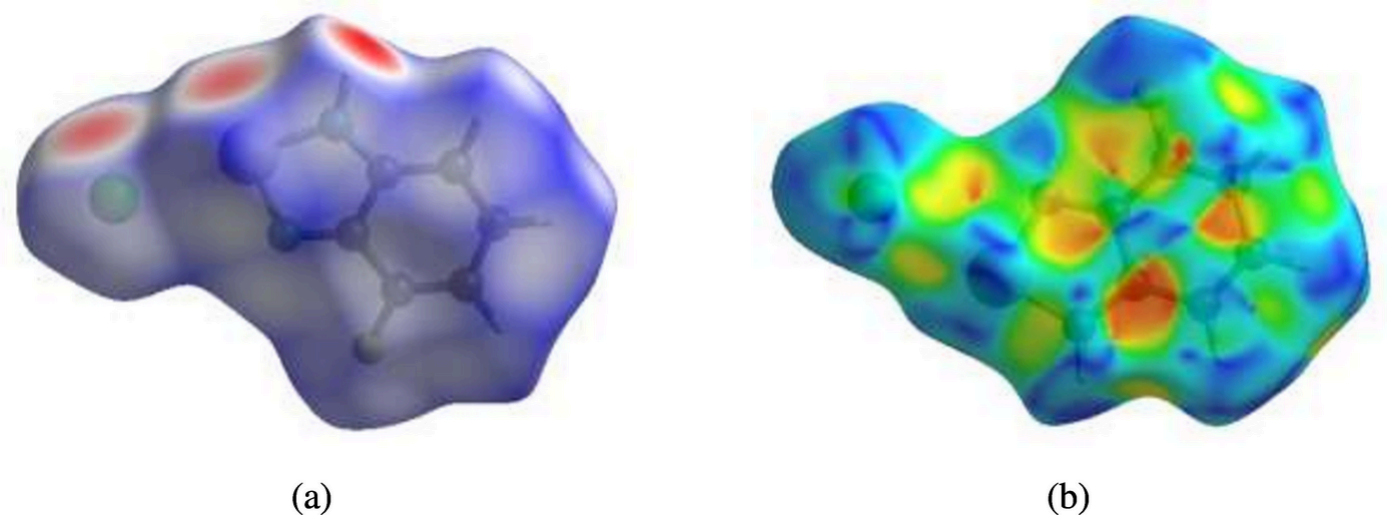
(*a*) View of the three-dimensional Hirshfeld surface of the title compound plotted over *d*_norm_ and (*b*) Hirshfeld surface of the title compound plotted over shape-index.

A Hirshfeld surface (HS) analysis (Hirshfeld, 1977 ▸; Spackman & Jayatilaka, 2009 ▸) was carried out to visualize the inter­molecular inter­actions in the crystal of the title compound using *CrystalExplorer* (Spackman *et al.*, 2021 ▸). In the three-dimensional Hirshfeld surface plotted over *d*_norm_ (Fig. 3 ▸*a*), the contact distances equal to the sum of van der Waals radii are shown by the white surfaces, whereas distances shorter and longer than the van der Waals radii are shown in red and blue, respectively (Venkatesan *et al.*, 2016 ▸), where the bright-red spots indicate their roles as the respective donors and/or acceptors. Planar stacking arrangements and the presence of aromatic stacking inter­actions such as C—H⋯π and π–π inter­actions are visualized by shape-index. In the HS plotted over shape-index, the C—H⋯π inter­actions are represented as red π-holes, which are related to the electron ring inter­actions between the CH groups with the centroid of the aromatic rings of neighbouring mol­ecules. On the other hand, π–π stacking inter­actions are visualized by the presence of adjacent red and blue triangles. Fig. 3 ▸*b* clearly suggests that there are neither C—H⋯π nor π–π inter­actions present.

**Figure 4:**
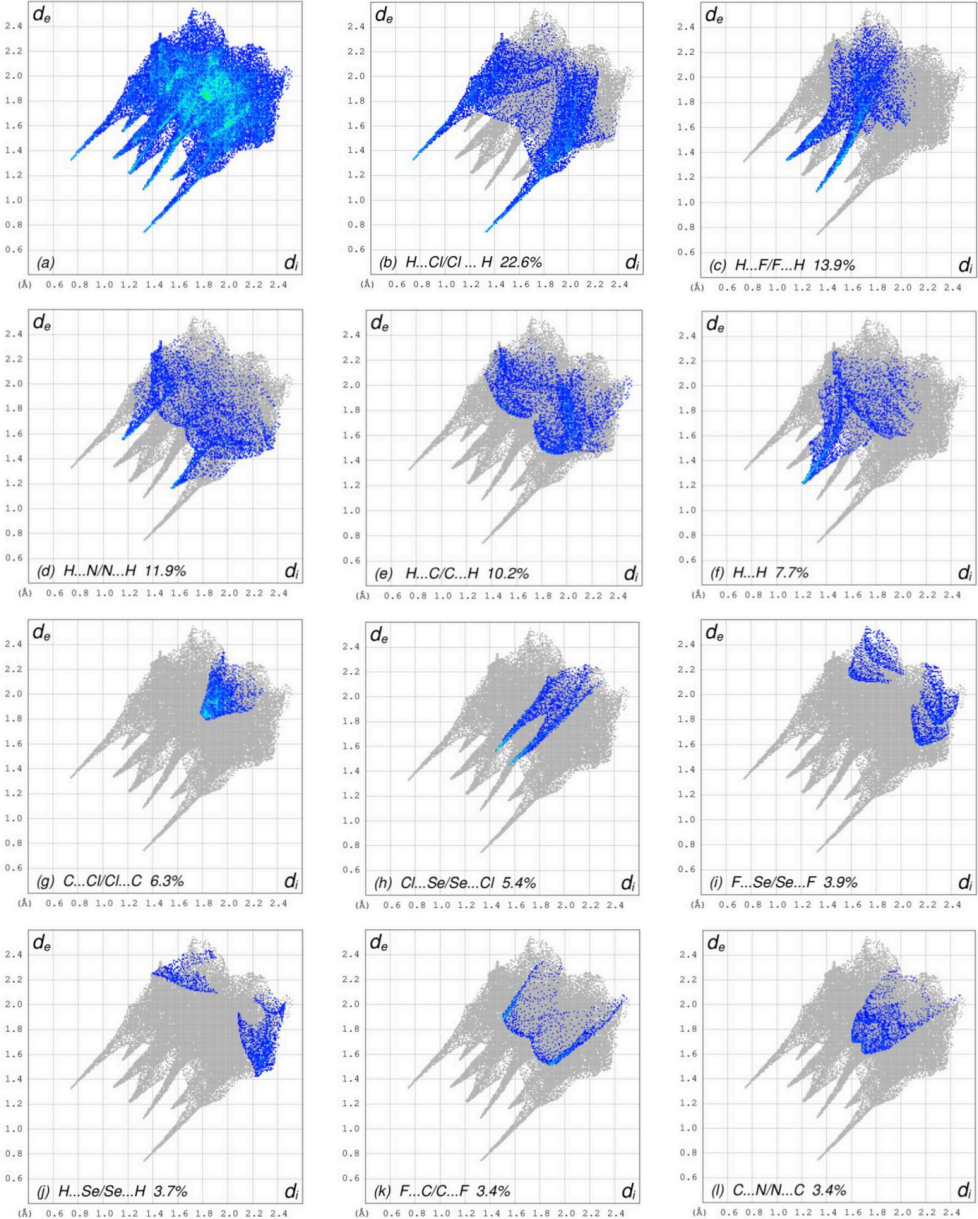
The full two-dimensional fingerprint plots for the title compound, showing (*a*) all inter­actions, and delineated into (*b*) H⋯Cl/Cl⋯H, (*c*) H⋯F/F⋯H, (*d*) H⋯N/N⋯H, (*e*) H⋯C/C⋯H, (*f*) H⋯H, (*g*) C⋯Cl/Cl⋯C, (*h*) Cl⋯Se/Se⋯Cl, (i) F⋯Se/Se⋯F, (*j*) H⋯Se/Se⋯H, (*k*) F⋯C/C⋯F, (*l*) C⋯N/N⋯C, (*m*) N⋯Se/Se⋯N, (*n*) C⋯Se/Se⋯C, (*o*) F⋯Cl/Cl⋯F, (*p*) N⋯N, (*q*) C⋯C, (*r*) Se⋯Se and (*s*) F⋯N/N⋯F inter­actions. The *d*_i_ and *d*_e_ values are the closest inter­nal and external distances (in Å) from given points on the Hirshfeld surface.

The overall two-dimensional fingerprint plot, Fig. 4 ▸*a*, and those delineated into H⋯Cl/Cl⋯H, H⋯F/F⋯H, H⋯N/N⋯H, H⋯C/C⋯H, H⋯H, C⋯Cl/Cl⋯C, Cl⋯Se/Se⋯Cl, F⋯Se/Se⋯F, H⋯Se/Se⋯H, F⋯C/C⋯F, C⋯N/N⋯C, N⋯Se/Se⋯N, C⋯Se/Se⋯C, F⋯Cl/Cl⋯F, N⋯N, C⋯C, Se⋯Se and F⋯N/N⋯F (McKinnon *et al.*, 2007 ▸) are illustrated in Fig. 4 ▸*b–s*, respectively, together with their relative contributions to the Hirshfeld surface. The most important inter­action is H⋯Cl/Cl⋯H (Fig. 4 ▸*b*), contributing 22.6% to the HS, and viewed as a pair of spikes at *d*_e_ + *d*_i_ = 2.06 Å. The H⋯F/F⋯H (Table 2 ▸ and Fig. 4 ▸*c*) and H⋯N/N⋯H (Fig. 4 ▸*d*) contacts contribute 13.9% and 11.9%, respectively, to the HS and are viewed as pairs of spikes at *d*_e_ + *d*_i_ = 2.42 Å and *d*_e_ + *d*_i_ = 2.72 Å, respectively. In the absence of C—H⋯π inter­actions, the H⋯C/C⋯H contacts (Fig. 4 ▸*e*), contributing 10.2% to the HS, are reflected at *d*_e_ + *d*_i_ = 3.28 Å. The H⋯H inter­actions (Fig. 4 ▸*f*) contribute 7.7% to the HS, and are viewed at *d*_e_ = *d*_i_ = 1.22 Å. The C⋯Cl/Cl⋯C contacts (Fig. 4 ▸*g*) with a 6.3% contribution to the HS, have an arrow-shaped distribution of points, and they are viewed at *d*_e_ = *d*_i_ = 1.84 Å. The pair of spikes of the Cl⋯Se/Se⋯Cl contacts (Fig. 4 ▸*h*) with 5.4% contribution to the HS are seen at *d*_e_ + *d*_i_ = 3.00 Å. Finally, the F⋯Se/Se⋯F (Fig. 4 ▸*i*), H⋯Se/Se⋯H (Fig. 4 ▸*j*), F⋯C/C⋯F (Fig. 4 ▸*k*), C⋯N/N⋯C (Fig. 4 ▸*l*), N⋯Se/Se⋯N (Fig. 4 ▸*m*), C⋯Se/Se⋯C (Fig. 4 ▸*n*), F⋯Cl/Cl⋯F (Fig. 4 ▸*o*), N⋯N (Fig. 4 ▸*p*), C⋯C (Fig. 4 ▸*q*), Se⋯Se (Fig. 4 ▸*r*) and F⋯N/N⋯F (Fig. 4 ▸*s*) contacts with 3.9%, 3.7%, 3.4%, 3.4%, 2.1%, 1.4%, 1.2%, 1.1%, 1.1%, 0.5% ad 0.2% contributions, respectively, to the HS make very small contributions.

**Figure 5:**
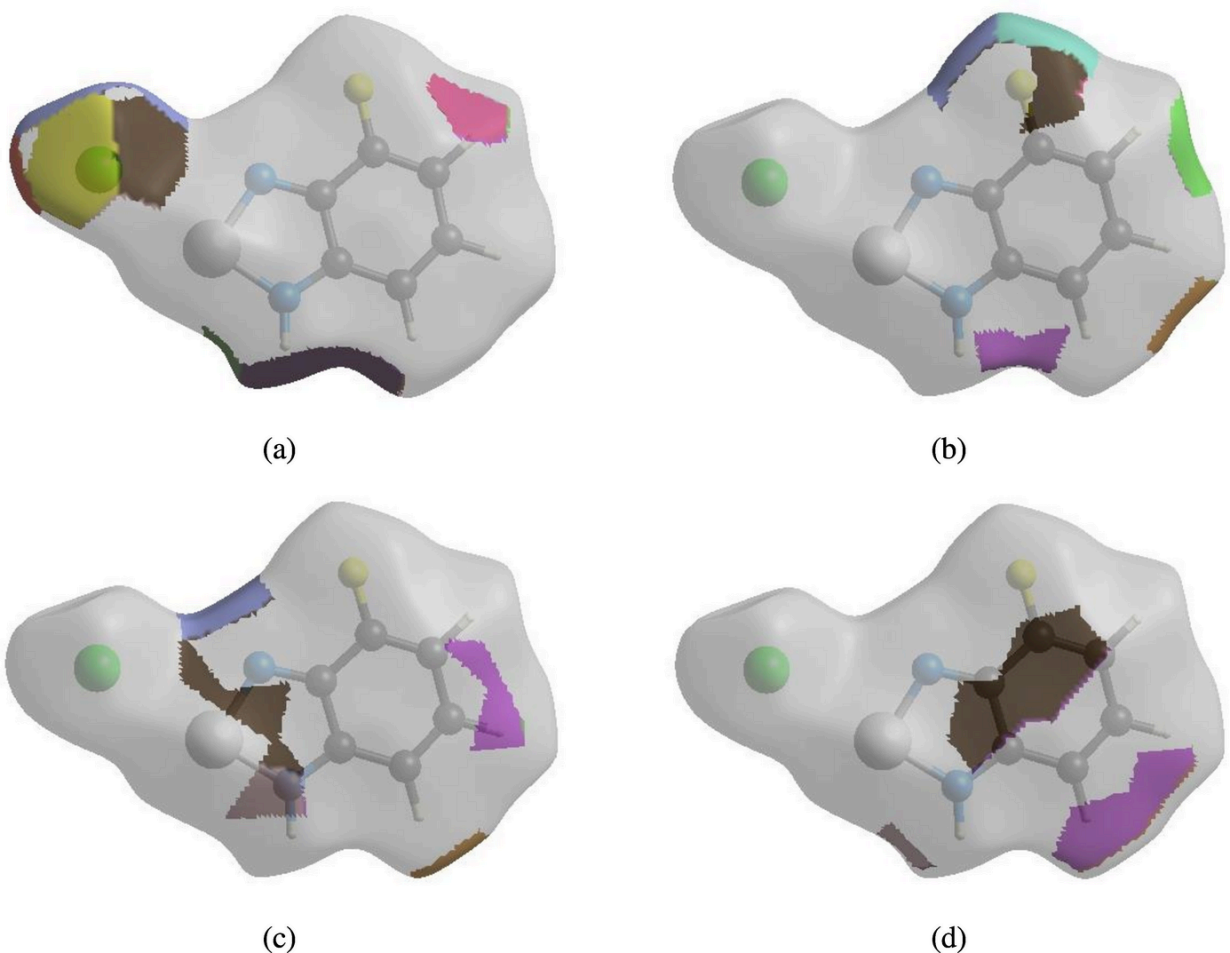
The Hirshfeld surface representations with the function *d*_norm_ plotted onto the surface for (*a*) H⋯Cl/Cl⋯H, (*b*) H⋯F/F⋯H, (*c*) H⋯N/N⋯H and (*d*) H⋯C/C⋯H inter­actions.

The nearest neighbour coordination environment of a mol­ecule can be determined from the colour patches on the HS based on how close to other mol­ecules they are. The HS representations of contact patches plotted onto the surface are shown for the H⋯Cl/Cl⋯H, H⋯F/F⋯H, H⋯N/N⋯H and H⋯C/C⋯H inter­actions in Fig. 5 ▸*a–d*.

The Hirshfeld surface analysis confirms the importance of H-atom contacts in establishing the packing. The large number of H⋯Cl/Cl⋯H, H⋯F/F⋯H, H⋯N/N⋯H and H⋯C/C⋯H inter­actions suggest that van der Waals inter­actions and hydrogen bonding play the major roles in the crystal packing (Hathwar *et al.*, 2015 ▸).

## 5. Crystal voids

**Figure 6:**
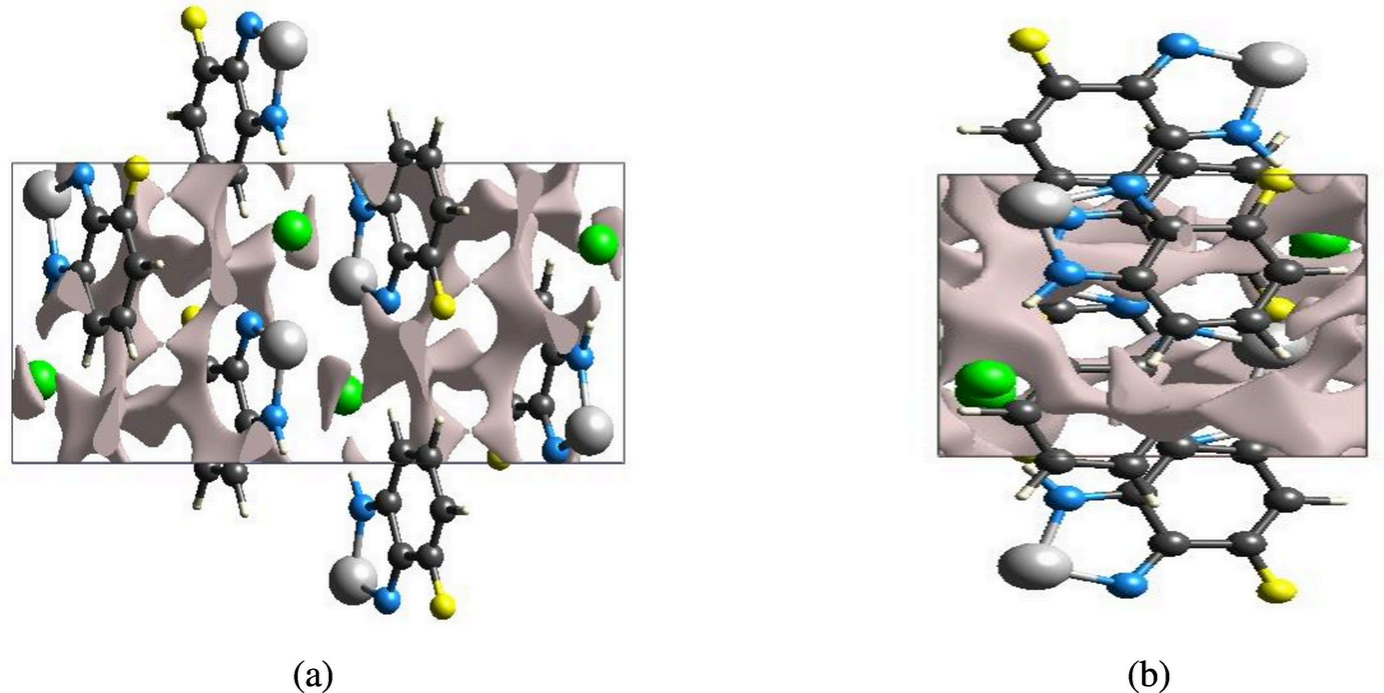
Graphical views of voids in the crystal packing of the title compound (*a*) along the *a* axis and (*b*) along the *b* axis.

If the crystal packing does not result in significant voids, then the mol­ecules are tightly packed and the applied external mechanical force may not easily break the crystal. Thus, the strength of the crystal packing is important for determining the response to an applied mechanical force. To check the mechanical stability of the crystal, a void analysis was performed by adding up the electron densities of the spherically symmetric atoms contained in the asymmetric unit (Turner *et al.*, 2011 ▸; Irrou *et al.*, 2022 ▸). The volume of the crystal voids (Fig. 6 ▸*a*,*b*) and the percentage of free space in the unit cell were calculated to be 44.80 Å^3^ and 5.91%, respectively. Thus, the crystal packing appears compact and the mechanical stability should be substantial.

## 6. Database survey

A survey of the Cambridge Structural Database (CSD; version 5.45, update of September 2024; Groom *et al.*, 2016 ▸) found two mol­ecules that are similar to the title compound, *viz*. (*rac*)-4-methyl-4-nitro-2,1,3-benzoselena-diazol-5(4*H*)-one, C_7_H_5_N_3_O_3_Se (CSD refcode JURLAJ; Tian *et al.*, 1993 ▸) and 5-nitro-2,1,3-benzoselena­diazole, C_6_H_3_N_3_O_2_Se (CSD refcode DOBWUO; Aliyeva *et al.*, 2024 ▸). In contrast to the four-membered Se_2_Cl_2_ ring defined through charge-assisted chalcogen bonds in the crystal packing of the title compound, there is an Se_2_N_*2*_ supra­molecular synthon with inter­molecular chalcogen bonds in JURLAJ and DOBWUO.

## 7. Synthesis and crystallization

3-Fluoro­benzene-1,2-di­amine (10 mmol) and selenium dioxide (10 mmol) were dissolved in 25 ml of di­chloro­methane and stirred for 1 h at ambient temperature, and further refluxed for 1 h. After cooling to room temperature, the solvent was evaporated under reduced pressure to give the reaction product. The title compound was obtained by slow evaporation of a water–acetone (1:3 *v*:*v*) solution of the reaction product at pH = 2 (adjusted by addition of HCl), and analysed by single-crystal X-ray analysis, elemental analysis, ESI-MS and NMR measurements. Yield: 87% (based on SeO_2_), yellow powder soluble in methanol, ethanol and DMSO. Analysis calculated for C_6_H_4_ClFN_2_Se (*M*_r_ = 237.53): C, 30.34; H, 1.70; N, 11.79. Found: C, 30.29; H, 1.67; N, 11.76. ESI-MS (positive ion mode), *m*/*z*: 238.4 [*M* + H]^+. 1^H NMR (CDCl_3_), δ: 6.77–7.92 (3H, Ar–H). ^13^C NMR (CDCl_3_), 110.91, 119.58, 128.96, 151.29, 155.67 and 161.93.

## 8. Refinement

Crystal data, data collection and structure refinement details are summarized in Table 3 ▸. The N- and C-bond hydrogen atom positions were calculated geometrically at distances of 0.85 Å and 0.93 Å (for aromatic CH) and refined using a riding model by applying the constraint of *U*_iso_(H) = 1.2*U*_eq_(C, N).

## Supplementary Material

Crystal structure: contains datablock(s) I. DOI: 10.1107/S2056989025001379/wm5748sup1.cif

Structure factors: contains datablock(s) I. DOI: 10.1107/S2056989025001379/wm5748Isup2.hkl

Supporting information file. DOI: 10.1107/S2056989025001379/wm5748Isup3.cml

CCDC reference: 2424642

Additional supporting information:  crystallographic information; 3D view; checkCIF report

## Figures and Tables

**Table 1:** Hydrogen-bond geometry (Å, °)

*D*—H⋯*A*	*D*—H	H⋯*A*	*D*⋯*A*	*D*—H⋯*A*
N2—H2*N*⋯Cl1^i^	0.85	2.23	3.056 (3)	163

Symmetry code: (i) 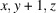.

**Table 2:** Selected interatomic distances (Å)

N2⋯Cl1^i^	3.056 (3)	F1⋯N1	2.800 (3)
H2*N*⋯Cl1^i^	2.23	H5⋯F1^ii^	2.56

Symmetry codes: (i) 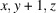; (ii) 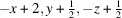.

**Table 3:** Experimental details

Crystal data	
Chemical formula	C_6_H_4_FN_2_Se^+^·Cl^−^
*M*_r_	237.52
Crystal system, space group	Monoclinic, *P*2_1_/*c*
Temperature (K)	296
*a*, *b*, *c* (Å)	6.889 (4), 7.250 (5), 15.183 (10)
β (°)	90.90 (3)
*V* (Å^3^)	758.2 (9)
*Z*	4
Radiation type	Mo *K*α
μ (mm^−1^)	5.25
Crystal size (mm)	0.34 × 0.23 × 0.14

Data collection	
Diffractometer	Bruker APEXII CCD
Absorption correction	Multi-scan (*SADABS*; Krause *et al.*, 2015 ▸)
*T*_min_, *T*_max_	0.252, 0.498
No. of measured, independent and observed [*I* > 2σ(*I*)] reflections	9806, 1768, 1528
*R*_int_	0.046
(sin θ/λ)_max_ (Å^−1^)	0.658

Refinement	
*R*[*F*^2^ > 2σ(*F*^2^)], *wR*(*F*^2^), *S*	0.025, 0.055, 1.07
No. of reflections	1768
No. of parameters	100
H-atom treatment	H-atom parameters constrained
Δρ_max_, Δρ_min_ (e Å^−3^)	0.27, −0.41

Computer programs: *APEX4* and *SAINT* (Bruker, 2020 ▸), *SHELXT2019/1* (Sheldrick, 2015*a* ▸), *SHELXL2019/1* (Sheldrick, 2015*b* ▸), *SHELXTL* (Sheldrick, 2008 ▸) and *publCIF* (Westrip, 2010 ▸).

## supplementary crystallographic information

### 4-Fluorobenzo[c][1,2,5]selenadiazol-1-ium chloride Crystal data

**Table:**

C_6_H_4_FN_2_Se^+^·Cl^−^	*F*(000) = 456
*M**_r_* = 237.52	*D*_x_ = 2.081 Mg m^−^^3^
Monoclinic, *P*2_1_/*c*	Mo *K*α radiation, λ = 0.71073 Å
*a* = 6.889 (4) Å	Cell parameters from 3456 reflections
*b* = 7.250 (5) Å	θ = 3.0–27.8°
*c* = 15.183 (10) Å	µ = 5.25 mm^−^^1^
β = 90.90 (3)°	*T* = 296 K
*V* = 758.2 (9) Å^3^	Plate, yellow
*Z* = 4	0.34 × 0.23 × 0.14 mm

### 4-Fluorobenzo[c][1,2,5]selenadiazol-1-ium chloride Data collection

**Table:**

Bruker APEXII CCD diffractometer	1528 reflections with *I* > 2σ(*I*)
φ and ω scans	*R*_int_ = 0.046
Absorption correction: multi-scan (SADABS; Krause *et al.*, 2015)	θ_max_ = 27.9°, θ_min_ = 3.0°
*T*_min_ = 0.252, *T*_max_ = 0.498	*h* = −8→9
9806 measured reflections	*k* = −8→9
1768 independent reflections	*l* = −19→19

### 4-Fluorobenzo[c][1,2,5]selenadiazol-1-ium chloride Refinement

**Table:**

Refinement on *F*^2^	Primary atom site location: structure-invariant direct methods
Least-squares matrix: full	Secondary atom site location: difference Fourier map
*R*[*F*^2^ > 2σ(*F*^2^)] = 0.025	Hydrogen site location: mixed
*wR*(*F*^2^) = 0.055	H-atom parameters constrained
*S* = 1.07	*w* = 1/[σ^2^(*F*_o_^2^) + (0.0085*P*)^2^ + 0.4749*P*] where *P* = (*F*_o_^2^ + 2*F*_c_^2^)/3
1768 reflections	(Δ/σ)_max_ = 0.001
100 parameters	Δρ_max_ = 0.27 e Å^−^^3^
0 restraints	Δρ_min_ = −0.41 e Å^−^^3^

### 4-Fluorobenzo[c][1,2,5]selenadiazol-1-ium chloride Special details

**Table:**

Geometry. All esds (except the esd in the dihedral angle between two l.s. planes) are estimated using the full covariance matrix. The cell esds are taken into account individually in the estimation of esds in distances, angles and torsion angles; correlations between esds in cell parameters are only used when they are defined by crystal symmetry. An approximate (isotropic) treatment of cell esds is used for estimating esds involving l.s. planes.

### 4-Fluorobenzo[c][1,2,5]selenadiazol-1-ium chloride Fractional atomic coordinates and isotropic or equivalent isotropic displacement parameters (Å^2^)

**Table:**

	*x*	*y*	*z*	*U*_iso_*/*U*_eq_	
Se1	0.22554 (3)	0.60768 (4)	0.44282 (2)	0.03658 (9)	
Cl1	0.10964 (9)	0.22691 (9)	0.45729 (5)	0.04730 (16)	
F1	0.7896 (2)	0.5196 (3)	0.29631 (12)	0.0683 (5)	
N1	0.4403 (3)	0.5410 (3)	0.38754 (13)	0.0369 (4)	
N2	0.2954 (3)	0.8495 (3)	0.42793 (13)	0.0369 (4)	
H2N	0.224535	0.941205	0.440922	0.044*	
C1	0.5393 (3)	0.6891 (4)	0.36440 (14)	0.0341 (5)	
C2	0.4619 (3)	0.8681 (3)	0.38569 (14)	0.0345 (5)	
C3	0.5575 (3)	1.0345 (4)	0.36309 (16)	0.0430 (6)	
H3	0.506553	1.149204	0.377483	0.052*	
C4	0.7274 (4)	1.0179 (5)	0.31933 (17)	0.0502 (7)	
H4	0.794289	1.124494	0.304325	0.060*	
C5	0.8070 (4)	0.8445 (5)	0.29559 (18)	0.0521 (7)	
H5	0.922383	0.840209	0.264667	0.063*	
C6	0.7178 (3)	0.6873 (4)	0.31723 (16)	0.0434 (6)	

### 4-Fluorobenzo[c][1,2,5]selenadiazol-1-ium chloride Atomic displacement parameters (Å^2^)

**Table:**

	*U*^11^	*U*^22^	*U*^33^	*U*^12^	*U*^13^	*U*^23^
Se1	0.03476 (13)	0.02831 (14)	0.04693 (14)	0.00279 (9)	0.00965 (8)	−0.00143 (11)
Cl1	0.0487 (3)	0.0279 (3)	0.0659 (4)	0.0022 (2)	0.0187 (3)	−0.0004 (3)
F1	0.0536 (9)	0.0656 (14)	0.0865 (12)	0.0207 (9)	0.0249 (8)	−0.0028 (11)
N1	0.0354 (9)	0.0326 (12)	0.0427 (10)	0.0062 (8)	0.0051 (8)	−0.0022 (9)
N2	0.0378 (9)	0.0262 (12)	0.0469 (11)	0.0033 (8)	0.0091 (8)	−0.0032 (9)
C1	0.0312 (10)	0.0345 (14)	0.0367 (11)	0.0027 (9)	0.0008 (8)	−0.0027 (10)
C2	0.0354 (11)	0.0332 (14)	0.0347 (11)	−0.0019 (9)	−0.0008 (8)	−0.0001 (10)
C3	0.0460 (12)	0.0352 (15)	0.0477 (13)	−0.0055 (11)	−0.0009 (10)	0.0023 (12)
C4	0.0463 (13)	0.053 (2)	0.0509 (15)	−0.0145 (12)	0.0026 (11)	0.0094 (13)
C5	0.0367 (12)	0.066 (2)	0.0536 (15)	−0.0020 (12)	0.0100 (10)	0.0076 (15)
C6	0.0359 (11)	0.0482 (18)	0.0462 (14)	0.0078 (11)	0.0066 (10)	−0.0006 (12)

### 4-Fluorobenzo[c][1,2,5]selenadiazol-1-ium chloride Geometric parameters (Å, º)

**Table:**

Se1—N1	1.779 (2)	C2—C3	1.419 (4)
Se1—N2	1.833 (2)	C3—C4	1.360 (4)
F1—C6	1.352 (4)	C3—H3	0.9300
N1—C1	1.322 (3)	C4—C5	1.420 (5)
N2—C2	1.330 (3)	C4—H4	0.9300
N2—H2N	0.8500	C5—C6	1.339 (4)
C1—C6	1.433 (3)	C5—H5	0.9300
C1—C2	1.442 (4)		
			
N2···Cl1^i^	3.056 (3)	F1···N1	2.800 (3)
H2N···Cl1^i^	2.23	H5···F1^ii^	2.56
			
N1—Se1—N2	88.83 (10)	C4—C3—H3	121.7
C1—N1—Se1	109.94 (18)	C2—C3—H3	121.7
C2—N2—Se1	112.75 (17)	C3—C4—C5	122.8 (3)
C2—N2—H2N	122.4	C3—C4—H4	118.6
Se1—N2—H2N	124.5	C5—C4—H4	118.6
N1—C1—C6	125.1 (2)	C6—C5—C4	120.7 (2)
N1—C1—C2	118.5 (2)	C6—C5—H5	119.6
C6—C1—C2	116.3 (2)	C4—C5—H5	119.6
N2—C2—C3	127.6 (2)	C5—C6—F1	122.5 (2)
N2—C2—C1	110.0 (2)	C5—C6—C1	121.1 (3)
C3—C2—C1	122.5 (2)	F1—C6—C1	116.5 (3)
C4—C3—C2	116.7 (3)		
			
N2—Se1—N1—C1	0.14 (16)	N2—C2—C3—C4	−179.8 (2)
N1—Se1—N2—C2	0.13 (17)	C1—C2—C3—C4	0.4 (3)
Se1—N1—C1—C6	−178.75 (19)	C2—C3—C4—C5	0.8 (4)
Se1—N1—C1—C2	−0.4 (3)	C3—C4—C5—C6	−1.2 (4)
Se1—N2—C2—C3	179.83 (19)	C4—C5—C6—F1	−179.6 (2)
Se1—N2—C2—C1	−0.3 (2)	C4—C5—C6—C1	0.4 (4)
N1—C1—C2—N2	0.5 (3)	N1—C1—C6—C5	179.2 (2)
C6—C1—C2—N2	179.0 (2)	C2—C1—C6—C5	0.8 (4)
N1—C1—C2—C3	−179.7 (2)	N1—C1—C6—F1	−0.9 (4)
C6—C1—C2—C3	−1.2 (3)	C2—C1—C6—F1	−179.31 (19)

Symmetry codes: (i) *x*, *y*+1, *z*; (ii) −*x*+2, *y*+1/2, −*z*+1/2.

### 4-Fluorobenzo[c][1,2,5]selenadiazol-1-ium chloride Hydrogen-bond geometry (Å, º)

**Table:**

*D*—H···*A*	*D*—H	H···*A*	*D*···*A*	*D*—H···*A*
N2—H2*N*···Cl1^i^	0.85	2.23	3.056 (3)	163

Symmetry code: (i) *x*, *y*+1, *z*.
